# Effect of body size on heat tolerance of a freshwater catfish (*Trichomycterus areolatus*)

**DOI:** 10.1093/conphys/coaf081

**Published:** 2025-11-25

**Authors:** Daniel Avilés-Hernández, Cristián A Zamora, Ian Calderon-Castro, D Patricio Carrizo, Gustavo Chiang, Enrico L Rezende, Mauricio J Carter

**Affiliations:** One Health Institute, Facultad de Ciencias de la Vida, Universidad Andrés Bello, Republica 440, 8370251, Santiago, Chile; Center of Applied Ecology and Sustainability (CAPES), Facultad de Ciencias Biológicas, Universidad Católica de Chile, Av. Libertador Bernardo O’Higgins 340, 8320000, Santiago , Chile; Center of Applied Ecology and Sustainability (CAPES), Facultad de Ciencias Biológicas, Universidad Católica de Chile, Av. Libertador Bernardo O’Higgins 340, 8320000, Santiago , Chile; One Health Institute, Facultad de Ciencias de la Vida, Universidad Andrés Bello, Republica 440, 8370251, Santiago, Chile; One Health Institute, Facultad de Ciencias de la Vida, Universidad Andrés Bello, Republica 440, 8370251, Santiago, Chile; One Health Institute, Facultad de Ciencias de la Vida, Universidad Andrés Bello, Republica 440, 8370251, Santiago, Chile; Centro para la Resiliencia, Adaptación y Mitigación (CReAM), Universidad Mayor, 4801043, Temuco, Chile; Escuela de Agronomía, Facultad de Ciencias, Ingeniería y Tecnología, Universidad Mayor, 4780000, Temuco P.O. Box 54-D, Chile; Center of Applied Ecology and Sustainability (CAPES), Facultad de Ciencias Biológicas, Universidad Católica de Chile, Av. Libertador Bernardo O’Higgins 340, 8320000, Santiago , Chile; One Health Institute, Facultad de Ciencias de la Vida, Universidad Andrés Bello, Republica 440, 8370251, Santiago, Chile; Center of Applied Ecology and Sustainability (CAPES), Facultad de Ciencias Biológicas, Universidad Católica de Chile, Av. Libertador Bernardo O’Higgins 340, 8320000, Santiago , Chile

**Keywords:** Body mass, freshwater, hypoxia, thermal death time curves, thermal tolerance, *Trichomycterus areolatus*

## Abstract

Rivers are under intense anthropogenic pressure, leading to increases in water temperature and changes in physicochemical properties, which threaten aquatic biota. Understanding how these environmental changes affect heat tolerance in freshwater organisms is critical for assessing the status of wild populations and predicting their vulnerability under global warming scenarios. Here, we studied how body mass and heat tolerance, measured by thermal death time (TDTs) curves under normoxic and hypoxic conditions, vary among populations of the Chilean pencil catfish *Trichomycterus areolatus* inhabiting a Mediterranean river in central Chile. We detected significant differences in fork length, body mass and Fulton’s condition factor among populations, with fish from reference sites being significantly larger and in better condition. Although heat tolerance did not differ among populations, we found a strong effect of body mass under both normoxic and hypoxic experimental conditions. Simulations combining laboratory-derived TDTs with field-recorded water temperatures suggest that the window of vulnerability occurs at lower temperatures but over longer exposures, indicating that heat stress has chronic effects on *T. areolatus*. Accordingly, the cumulative survival simulation using the warmer season records is predicted to be lower in river sections with reduced levels of dissolved oxygen. While our results did not show population level differences in thermal tolerance *per se*, the significant effect of individual body mass may translate into varying vulnerability among populations, given their marked differences in body mass distribution. These findings highlight how the interplay between water quality, body condition and heat tolerance shapes the vulnerability of *T. areolatus* populations to warming. Thus, an integrated perspective is essential to properly assess the impact of global warming on wild freshwater populations.

## 1. Introduction

Anthropogenic activities are a major driver of change in freshwater ecosystems, profoundly impacting biodiversity (Woodward *et al*., 2010). Pollution and environmental degradation alter biological communities (Mehdi *et al*., 2022), especially for fish (Habit *et al*., 2006; Orrego *et al.*, 2009), while rising average temperatures driven by human activity (Kaushal *et al*., 2010; Liu *et al*., 2020) further amplify the effects of global warming (Moyer and Hawkins, 2017; Hobbie and Grimm, 2020). River temperatures are strongly influenced by atmospheric thermal conditions, riparian and geographical structures (Caissie, 2006), which enhances, e.g. the synergistic interaction of temperature with hypoxia (Earhart *et al*., 2022) or salinity (Ostrand and Wilde, 2001). Locally, rivers that traverse populated areas and urban heat islands are also expected to experience synergistic warming effects (Moyer and Hawkins, 2017). These factors contribute not only to a gradual, long-term increase in water temperature (Vasseur *et al*., 2014), but also to the occurrence of extreme events, such as heatwaves and thermal discharges from industrial activities, which can lead to acute thermal spikes that affect biological responses (Harris *et al*., 2018). In line with understanding the short- and long-term thermal stress (Harvey *et al*., 2022; Villeneuve and White, 2024), studies have documented the impacts of rising temperatures on riverine biodiversity and species distribution (Caissie, 2006; Troia and Giam, 2019).

Temperature critically shapes geographical and seasonal distribution of fish (Aigo *et al*., 2014). In response to heterogeneous thermal conditions across space and time, fish have evolved biochemical, physiological and behavioural adaptations, including thermal acclimation (Comte and Olden, 2017a), ontogenetic shifts (Dahlke *et al*., 2020) and local adaptation (Fangue *et al*., 2006; Villeneuve *et al*., 2021). Assessing the impact of heat stress on fish performance has been a challenging task, and researchers have historically employed different approaches, from ramping experiments to static thermal trials (Peck *et al*., 2009; Rezende *et al*., 2011; Molina *et al*., 2023). Thus, understanding the physiological and ecological mechanisms underlying variation in upper thermal limits across individuals, populations and species is crucial for predicting fish distributions and assessing warming tolerance (Campos *et al*., 2021; McKenzie *et al*., 2021; Molina *et al*., 2025). Additionally, the oxygen- and capacity-limited thermal tolerance (OCLTT) hypothesis (Pörtner, 2001) proposes that heat tolerance is constrained by oxygen availability, suggesting that variation among individuals may depend on physiology (Dillon *et al*., 2010), ontogeny (Illing *et al*., 2020) and/or different body size ranges (Leiva *et al*., 2019, 2024; Peralta-Maraver and Rezende, 2021). Indeed, recent studies on freshwater species such as amphipods have demonstrated that hypoxia can significantly impact heat tolerance and vulnerability to warming waters (Semsar-kazerouni *et al*., 2020; Verberk *et al*., 2023). Here, thermal death time (TDTs) curves leverage static protocols to assess time-dependent effects on thermal limits (Rezende *et al*., 2014), enabling differentiation between the time scale of heat stress response from acute (~minutes) and chronic (~hours in a daily scale) heat stress responses (Troia, 2023; Rezende and Carter, 2024).

Freshwater small-bodied fish generally have a restricted capacity to disperse within and between basins (Albert *et al*., 2011). Consequently, a close match is often observed between their physiological adaptations and the evolutionary history of the river basin. Fish from an area with levels of disturbance are affected in their individual performance, which impacts physiological parameters such as body condition and body mass (Merckx *et al*., 2018), as well as extirpation patterns, as a consequence of local stress and habitat selection may impact the population structure in the wild (Vander Vorste *et al*., 2020; Amat-Trigo *et al*., 2023). Additionally, the evidence suggests that South American freshwater fish may have a limited capacity for phenotypic acclimation to global warming (Campos *et al*., 2021), placing many species at risk of extirpation or extinction as temperatures rise worldwide (IPCC, 2021). This is particularly evident for fish inhabiting Chilean rivers, which are poorly connected given the Chilean geography, with river basins flowing from the Andes mountain range to the sea, and under pressure due to several human stressors (Orrego *et al.*, 2009; Chiang *et al*., 2011a).

**Figure 1:**
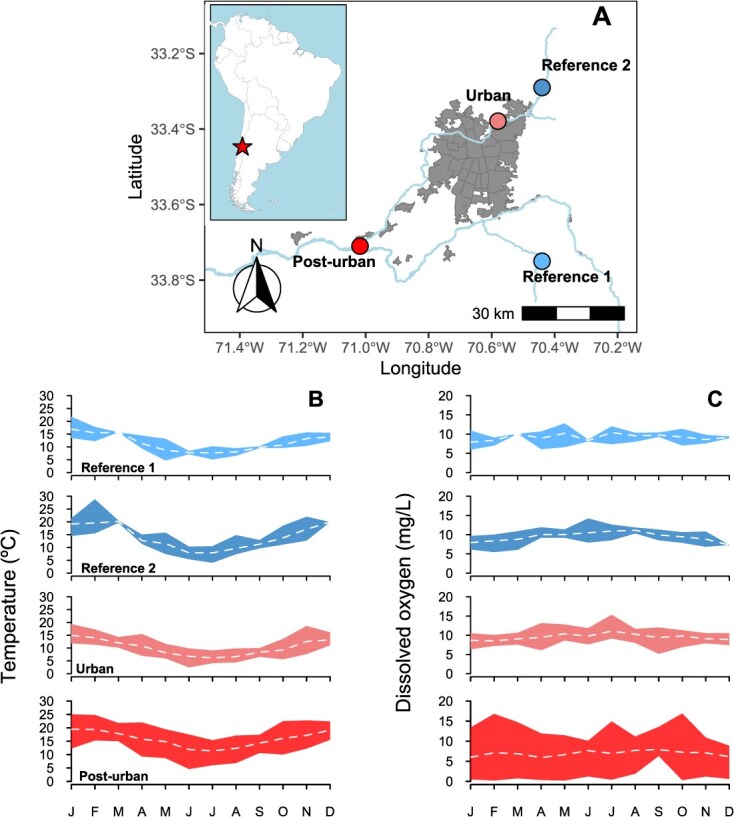
Sampling sites for body mass structure and TDT experiments across Maipo hydrological basin through the references sites (El Arrayan y El Clarillo), urban (Las Hualtatas) and post-urban (El Monte) areas, see Materials and Methods for details (**a**). Monthly temperature (**b**) and dissolved oxygen (**c**) profiles for four sampling sites. Data are presented as mean ± SD for data collection between January 1995 and December 2022. Data were accessed from the Chilean public governmental repository (DGA, see Material and Methods).

In this work, we studied a small-bodied fish model species *Trichomycterus areolatus*, a native pencil catfish species from the Maipo hydrological basin that crosses the city of Santiago (Fig. 1). The Maipo River Basin has suffered severe water quality degradation and eutrophication from untreated sewage (Fierro *et al*., 2019) and ranks among the most contaminated rivers worldwide (Jorquera *et al*., 2014; Wilkinson *et al*., 2022). These anthropogenic pressures have reduced fish richness and abundance (Habit *et al*., 2007), while diminishing genetic diversity in native species (Quezada-Romegialli *et al*., 2010; Vega-Retter *et al*., 2018). We hypothesize that urban-driven disturbances affect body size (fork length and mass) and warming tolerance in *T. areolatus* populations along an environmental degradation gradient. Specifically, we predict that populations from more impacted areas will exhibit smaller body sizes because of water quality loss, but greater heat tolerance compared to reference-site populations due to phenotypic selection or habitat extirpation. Then, by the implementation of a simple but powerful methodological approach (TDTs) and crossing with high-resolution thermal records to describe precisely the thermal habitat, we were able to model the cumulative mortality under contrasting thermal scenarios across different time scales with implications for predicting habitat suitability and fish spatial distribution.

## 2. Materials and Methods

### 2.1. Study organisms

The silurid *T. areolatus* is widely distributed throughout Chile, from 30° to 41° south latitude (Manriquez *et al*., 1988; Habit *et al*., 2006), inhabiting the riparian ecosystems along with several other fish species with narrower distribution (Alò *et al*., 2024), where also most of the anthropogenic development pressure is focused. Fish were obtained from different locations in Mapocho and Maipo Rivers (15.403 km^2^), which flow through one of the most populated urban areas in Chile (7 112 808 inhabitants in 2017, www.bcn.cl). Based on the absence of genetic differentiation previously reported for these populations (Quezada-Romegialli *et al*., 2010), we assume that *T. areolatus* sampled in the River Basin studies constitutes an interconnected metapopulation. Biological samples were collected from free-access riparian areas in Chilean rivers (executive order (DL) N ° 1.939–1977/BCN). The sampling survey was implemented in four locations (Fig. 1): two reference sites located before human disturbance at Río Clarillo (reference site 1), Estero El Arrayan (reference site 2), Río Mapocho-Las Hualtatas (urban site) and a countryside area at El Monte (post-urban site).

Individuals were captured during a one-week sampling period spanning late May to early June 2022, when water temperatures ranged between 9.6 and 17.1°C. We employed a backpack electrofishing unit (HT-2000, Halltech Environmental, Canada) and a block seine (6 mm mesh size) in riffles (0.2–0.3 m/s, 0.2–0.4 m depth) with boulder-cobble bedrock (~15 cm diameter) and/or shallow riffles (0.1–0.2 m/s, 0.1–0.4 m depth). For morphometric measurements, we identified, counted and measured fork length (±0.1 cm) and mass (±0.01 g) of all captured individuals before releasing some of them back to the river. We calculated the Fulton condition factor *K* = 100*(Mass (g)/Length^3^ (cm)) as a general indicator of physiological status (Ricker 1975). For laboratory heat tolerance trials, we selected a subset consisting exclusively of adult fish (60- to 110-mm-length range) from each sampling site (Chiang *et al*., 2012a). Since the animals were collected during the pre-reproductive period, we were unable to proceed with sex determination. Selected specimens were placed in a recirculation system maintained at 23°C for 1 week prior to experimental measurements.

Finally, as a proxy for water temperature variation experienced by fish in their natural habitat, we recorded warm-season temperatures (Austral summer 2021–2022) using three HOBO TidbiT v2 temperature sensors deployed at the Reference 1, Urban and Post-urban sampling sites. Additionally, to characterize long-term temperature and dissolved oxygen patterns at each site (Fig. 1b and c), we obtained ~35 years of historical records (Figure S1, Table S1) from the nearest monitoring stations reported by the *Dirección General de Agua* (DGA; https://snia.mop.gob.cl/BNAConsultas/reportes).

### 2.2. Heat tolerance estimates

To construct the TDT curves and quantify heat tolerance of *T. areolatus* populations, we randomly assigned subsampled individuals from each collection site to water baths maintained at 33, 33.5, 34 or 34.5°C. We tested 64 fish under normoxic conditions (8 mg O_2_/l), other 43 fish were used to build the TDTs in hypoxic conditions (4 mg O_2_/l). The range of oxygen dissolved concentration was chosen based on the reported range detected in the field (https://snia.mop.gob.cl/BNAConsultas/reportes). Oxygen concentration was manipulated by bubbling air or extra pure gaseous nitrogen (Verberk *et al*., 2023) and continuously monitored using a Microx optical fibre O_2_-meter (Presens Mini Oxy-4. Presens Inc., DE). During thermal trials, animals were placed inside an open jar in water bath with a heating coil (@Lauda) in a tank of 30 l. The fish were exposed to the stressful temperature until they presented signals of thermic stress (‘knockdown’), such as loss of equilibrium, defined as the inability of fish to maintain dorsoventral orientation for at least 1 min (Beitinger *et al*., 2000). Following trials, all individuals used were euthanized using an overdose of BZ20, following bioethics protocols of Universidad Andres Bello.

### 2.3. Statistical analysis

To identify the population structure in fork length, mass and condition factor (*K*), differences across sample sites were analyzed with an analysis of variance (ANOVA) followed by Tukey *post hoc* pairwise comparisons. From the experimental essay, we quantified heat tolerance using TDT curves to determine two key parameters: (i) thermal sensitivity (*z*) and (ii) critical thermal maximum (*CT*_max_). Thermal sensitivity (*z*) describes the relationship between thermal challenge intensity and exposure duration, calculated using the following linear equation:


(1) \begin{equation*} T_{\textrm{ko}}= CT_{\textrm{max}}-z\, log_{10}t \end{equation*}

where *T*_ko_ corresponds to the lethal temperature, *CT*_max_ to the ‘standard’ lethal temperature estimated for an exposure time of 1 min (i.e. the intercept when *log*_10_*t* equals 0) and *t* is the exposure time associated with each temperature (Rezende *et al*., 2014).

Then, we applied a model selection framework (Burnham and Anderson, 2002), treating measurement temperature (*T_ko_*), mass, contrasting dissolved oxygen conditions (4 and 8 mg O_2_/l) and sampling site as independent variables, and using the log10-transformed knockdown time for each fish as the dependent variable, structured as follows:


(2) \begin{equation*} \log_{10} t{\sim}T_{\textrm{ko}}+\log mass+ DO_{2}+site \end{equation*}

Models of increasing complexity were developed by sequentially incorporating independent variables and factors from left to right, along with all pairwise interactions (Carter *et al*., 2023). Higher order interactions were excluded to facilitate interpretation. The relative support for each model was evaluated using Akaike weights (wi), which indicate the probability that a given model is the best among all those tested. With this stepwise approach, we assessed how each factor, namely mass, and the sample sites, contributes to the knockdown time. For the model with the lowest Akaike Information Criterion with correction (AICc) values (i.e. the model with the best fit), we employed Cohen’s f to quantify the effect size and relative contribution of main effects and pairwise interactions.

Then, to model the potential impact of field hypoxic conditions on heat tolerance, we combined temperature and oxygenation data and calculated the effect of oxygen levels on the TDT curves obtained in the laboratory with the following linear model:


(3) \begin{equation*} \log_{10}t=\beta_{0}+\beta_{1}T_{\mathrm{ko}}+\beta_{2}\,D_{\mathrm{O}2}+\beta_{3}\,T_{\mathrm{ko}}\,D_{\mathrm{O}2}+\epsilon \end{equation*}

where *T_ko_* and *D*_O2_ correspond to the experimental temperatures (°C) and dissolved oxygen (mg O_2_/l). We calculated the parameters of the thermal-death curves [eq. (1)] as *CT*_max_ = −(*β*_0_ + *β*_2_  *D*_O2_)/(*β*_1_ + *β*_3_  *D*_O2_) and *z* = −1/(*β*_1_ + *β*_3_  *D*_O2_). This back-transformation is required when experiments control for *T_ko_* and measure collapse time as the dependent variable, otherwise the linear model will minimize the sums of squares and estimate parameters incorrectly (Rezende *et al*., 2014). We manually adjusted *D*_O2_ levels to generate the expected thermal tolerance landscape across different oxygen concentrations, enabling prediction of cumulative mortality under field-observed temperature fluctuations weighted by varying *D*_O2_ values. Following Rezende and Carter (2024), survival times were calculated employing eq. (2), to reconstruct the thermal tolerance landscape and then combine it with field temperature records to obtain the predicted mortality under these thermal conditions. These analyses are performed with *ad hoc* R functions *tolerance.landscape* and *mortality.isoclines* as detailed in their Supplementary Information. Additionally, we computed daily survival probabilities and cumulative survival across our entire temperature record for three distinct sites in the Maipo Basin. Based on the daily thermal fluctuation, we have defined that physiological recovery occurs during nocturnal temperature decreases to build our survival predictions. All analyses were conducted in R (https://www.R-project.org/).

**Table 1:** Summary of fish sizes from each of the four sample site populations. Fish were weighed and measured in the field during the sampling survey

Population	Sample size	Mass (g)	Fork length (cm)	Condition factor (*K*)
Reference site 1	47	2.13 ± 2.07	5.92 ± 2.11	0.87 ± 0.31
Reference site 2	62	3.03 ± 2.90	6.45 ± 2.50	0.84 ± 0.35
Urban site	51	3.36 ± 1.99	7.87 ± 1.75	0.61 ± 0.06
Post-urban site	80	1.57 ± 0.66	6.28 ± 0.95	0.61 ± 0.12
		*F*_(3,236)_ = 10.82 *P* < 0.001	*F*_(3,236)_ = 10.98 *P* < 0.001	*F*_(3,236)_ = 20.01 *P* < 0.001

Data are presented as mean ± SD. Fulton’s condition factor was calculated as *K* = 100 × (mass (g)/fork length^3^ (cm)). ANOVA tests are presented below every trait evaluated.

**Table 2:** Model comparison to test the impact of assay temperature *T*_ko_, body mass (*M*), *DO_2_* and sample sites on log-transformed knockdown time

Model	*K*	AIC_c_	ΔAIC_c_	*w_i_*	LogLik
log_10_ time *~ T*_ko_	3	176.3	121.4	0.00	−85.04
log_10_ time *~ T*_ko_ + log_10_ *M*	4	139.2	84.31	0.00	−65.37
log_10_ time *~ T*_ko_ + log_10_ *M* + *DO_2_*	5	67.59	12.74	0.00	−28.48
log_10_ time *~ T*_ko_ + log_10_ *M* + *DO_2_* + Site	8	71.02	16.16	0.00	−26.73
log_10_ time *~* (*T*_ko_ × log_10_ *M*)	5	136.5	81.71	0.00	−62.97
log_10_ time *~* (*T*_ko_ × *DO_2_)*	5	80.98	26.13	0.00	−35.17
log_10_ time *~* (*T*_ko_ × Site*)*	9	167.3	112.5	0.00	−73.69
**log**_**10**_ **time *~* (*T***_**ko**_ **+ log**_**10**_ ***M* + *DO***_***2***_**)**^**2**^	**8**	**54.85**	**0.00**	**1.00**	**−18.64**
log_10_ time *~* (*T*_ko_ + *DO_2_* + Site)^2^	14	91.30	36.45	0.00	29.21
log_10_ time *~* (*T*_ko_ + log_10_ *M* + Site)^2^	14	128.7	73.89	0.00	−47.93
log_10_ time *~* (*T*_ko_ + log_10_ *M* + *DO_2_* + Site)^2^	20	78.07	23.22	0.00	−13.79

The best model is shown in **bold.**

## 3. Results

### 3.1. Thermal death curves

We found significant differences in both fork length and mass of the catfish *T. areolatus* among sampling sites (Table 1). Pairwise comparisons in fork length indicate that fish from the urban site were smaller than the post-urban site, as well as that fish from the post-urban site were smaller than both reference site 1 and reference site 2 (Table S2). While comparison in mass indicated that fish from the Urban site were lighter than the post-urban site and reference site 1, likely for the post-urban and the reference site 2 (Table S2). Consequently, we found a significant difference in condition factor *K* in comparisons with fish from the urban site, which exhibited a lower *K* than both reference sites and a similar pattern to the post-urban site, with lower *K* compared with reference sites (Table S2).

**Figure 2:**
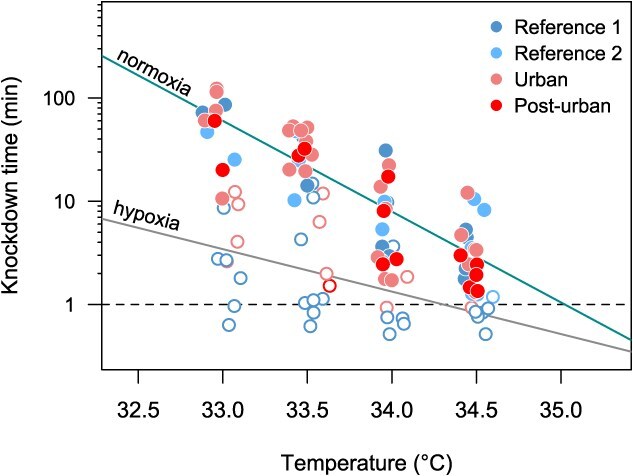
TDT curves built to estimate thermal sensitivity (*z*), and *CT*_max_ standardized at the same time scale in normoxic (~8 mgO_2_/l, solid points) and hypoxic experimental conditions (~4 mgO_2_/l, open points).

**Figure 3:**
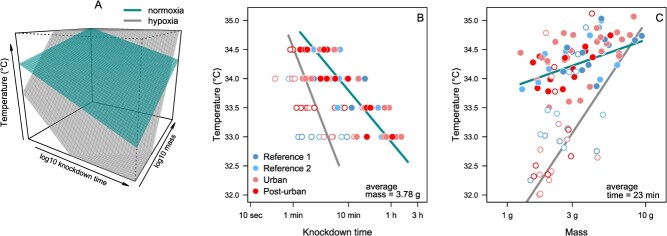
Predictions from the model the best fit of the response time to thermal stress. The 3D plot illustrates the positive association between mass and time, explaining knockdown time (*T*_ko_), split by normoxic and hypoxic conditions (**a**). Bi-dimensional prediction of the relation between average time response (**b**) and average body mass (**c**), both split by normoxic and hypoxic conditions.

Our model comparison approach revealed that the best model explaining the variation in knockdown time included mass and dissolved oxygen as predictors of the response to thermal stress (Table 2). Because the time of response for thermal stress did not vary among localities, we pooled the data to obtain a single TDT curve with *CT*_max_ = 35.0°C and a thermal sensitivity value of *z* = 1.14°C for normoxic (~8 mg O_2_/l) and *CT*_max_ = 34.3°C and *z* = 2.43°C for hypoxia (~4 mg O_2_/l) (Fig. 2). Partial regressions based on this model indicated that *T_ko_* increases with body mass (*F*_1,94_ = 112.13, *P* < 0.001), a pattern that is enhanced in fish exposed to hypoxic conditions (*F*_1,94_ = 12.5, *P* < 0.001) (Fig. 3).

### 3.2. Simulating mortality in the field

**Figure 4:**
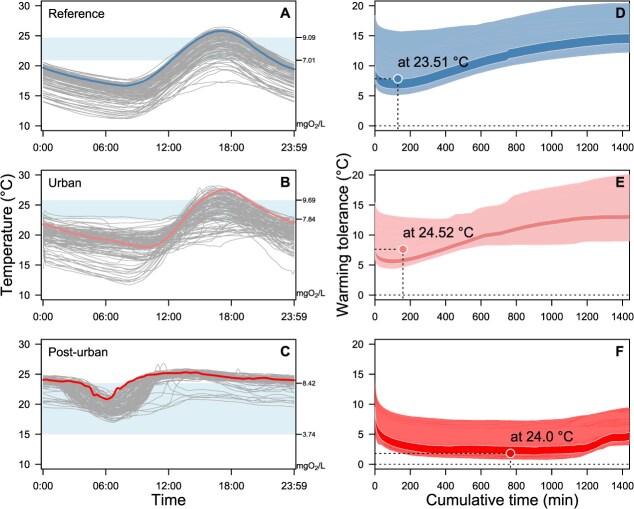
Daily thermal profile of water temperature of Maipo River from loggers deployed at the reference site 1 (**a**), urban site (**b**) and the post-urban site (**c**) data loggers records for warm season (188 days), and the range (Percentile 90–10) of dissolved oxygen variation in shade are obtained from the Chilean public governmental repository (DGA, see Materials and Methods). The polygon area presented daily warming vulnerability across the cumulative temperature as a projection considering the lower percentile (P_10_) of dissolved oxygen and mass range for the reference (**d**), urban (**e**) and post-urban (**f**) sites, respectively. Highlighted lines in daily record plots are mirrored in the corresponding polygons in cumulative mortality plots, and the average temperature at which lower vulnerability and cumulative time were estimated is indicated for each site.

**Figure 5:**
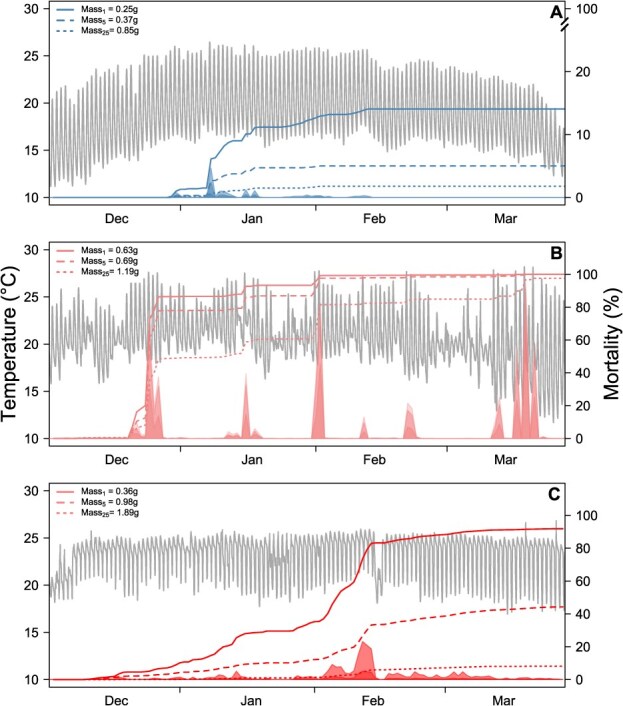
Cumulative survival was estimated across the temperature records from the warm season (grey lines), using the lower mass percentile (vulnerable sizes) represented by solid, dashed and dotted lines for a reference site population with a mean mass of 1.08 g (**a**), urban site with a mean mass of 1.47 g (**b**) and post-urban site with a mean mass of 1.23 g (**c**) sites, respectively. For illustrative purposes, cumulative survival was estimated with lower dissolved oxygen (P_10_) recorded in the post-urban site, which represents a realistic scenario that this freshwater system may experience.

Cumulative mortality was estimated using 118 days of highly detailed field records from three sites (see Table S3), which reflect the environmental contexts of the reference site 1, urban site and post-urban site. The analyses were performed to detect changes in warming tolerance at a daily scale (Fig. 4) and to calculate the cumulative mortality across the warmer season (Fig. 5), weighted by the variation in fish mass and the adjusted thermal sensitivity (*z*) at the P10 dissolved oxygen field records for each site. At a daily scale, the temperature patterns among sites impacted different populations in varying ways, affecting the warming tolerance across the mass distribution reported for each population. Reference site 1 presented a clear pattern among days with colder nights and warmer afternoons (Fig. 4a), with a range of 11.17–26.45°C. Using the mortality-isocline approach that allowed us to decompose the cumulative survival from different environmental factors, we found a lower warming tolerance was 7.8 ± 1.87°C (mean ± SD) estimated with a *z* = 7.01 across the 118, with a cumulative time of 129.7 ± 17.6 min (mean ± SD) and field record of 23.51 ± 1.89°C (mean ± SD) (Fig. 4e). A similar pattern was found to the urban site, though with more daily variation (Fig. 4b), with a range of 11.67–28.19°C. Here, the lower warming tolerance was 7.62 ± 1.84°C (mean ± SD) estimated with a *z* = 7.83, with a cumulative time of 158.6 ± 128.2 min (mean ± SD) and field record of 24.52 ± 2.02°C (mean ± SD) (Fig. 4f). A different pattern was found in the post-urban site, with a far more daily variation in comparison with the other sites (Fig. 4c), showing a range of 16.93–26.82°C. The lower warming tolerance was 1.81 ± 0.86°C (mean ± SD) estimated with a *z* = 3.74, with a cumulative time of 766.5 ± 188.9 min (mean ± SD) and field temperature of 24.0 ± 0.77°C (mean ± SD) (Fig. 4d). These analyses indicated that reduced daily mortality can still result in cumulative effects, and that sub-lethal temperature effects impacted fish performance. The simulations showed that thermal safety margins calculated on a realistic scale of variation (i.e. daily) captured the time dimension of the phenomena, as well as the variation in body mass, where larger fish are considerably less sensitive to water oxygenation changes (Fig. 4d–f).

To evidence the different patterns of cumulative mortality of *T. areolatus* through the warm season at three different river sites, we used the lower body mass class (Percentiles 1, 5 and 25), as well as the lower P_10_ of dissolved oxygen belonging to the Post-urban site, as the realistic example of hypoxia that Maipo–Mapocho river may experience. At the reference site, the cumulative mortality during the season to smaller fish was 12.8, 4.4 and 1.19% to P_1_, P_5_ and P_25_ of body mass, respectively (Fig. 5a). At the urban site 99.8% of the cumulative mortality was achieved in smaller fish body mass class (P_1_ and P_5_) before the season finish, while 96.0% was achieved for body mass class P25 (Fig. 5b). At the Post-urban site 96.5% of cumulative mortality was achieved in smaller body mass (P_1_), as well as 29.6% (P_5_) and 12.4% (P_25_), respectively, at the end of the season (Fig. 5c). The distinctive pattern of cumulative mortality over the time considered in the simulation results from differences in field temperature variation among study sites and in body mass among populations, indicating that smaller fish are more sensitive to heat stress.

## 4. Discussion

Determining how temperature variation affects thermal tolerance and identifying the mechanisms that modulate acclimatization and, ultimately, adaptation are primary targets of thermal ecologists (Comte and Olden, 2017b). Our results showed that variation in heat tolerance across individuals of *T. areolatus* with different levels of condition from the Maipo River Basin is influenced primarily by differences in body mass. Although we did not find significant differences between sampling sites, the differences in body mass distribution observed within and across localities, as well as the variation in dissolved oxygen, may impact mortality rates along different sections of the river under different warming scenarios (Figs 4 and 5). Overall, our findings suggest that heat stress might constitute a risk for *T. areolatus* in the context of global warming and that this effect may be mediated by water quality and its impact on body condition. This way, by revealing that chronic heat stress has a greater impact than acute effects, our methodological and analytical approach clarifies how physiological responses determine the long-term survival and conservation status of freshwater species.

The most significant finding of this work is the contribution of body mass to heat tolerance variation in *T. areolatus* individuals, though the underlying mechanisms remain speculative. Integrative traits like body mass may play a key role in mediating physiological processes that determine thermal performance (Pauly, 2021) with documented effects on heat tolerance across multiple taxa (Jørgensen *et al.*, 2021; Peralta-Maraver and Rezende, 2021) However, this pattern is not universal, as recent intrapopulation studies in fish have reported no body mass effect on heat tolerance (Gomez Isaza and Rodgers, 2024). While temperature increases have been generally linked to declining animal body size under global warming (Gardner *et al*., 2011; Lefevre *et al*., 2017), aquatic ectotherms show mixed responses (Audzijonyte *et al*., 2020; Lindmark *et al*., 2022; Letcher *et al*., 2023). Scaling changes in gill surface ratio (Rubalcaba *et al*., 2020; Pauly, 2021) have been proposed to explain body size associations with oxygen limitation (Leiva *et al*., 2019; Peralta-Maraver and Rezende, 2021; Verberk *et al*., 2022) and size dependency of hypoxia susceptibility (Leiva *et al*., 2018, 2024). Regardless of the mechanisms driving heat tolerance differences across size groups (Figs 4 and 5), the ecological implications are substantial. Body size distribution influences key demographic parameters including energy requirements, growth rates, mortality and recruitment, while allometric effects on heat tolerance may scale up to affect community structure and ecosystem functioning (Fritschie and Olden, 2016; Audzijonyte *et al*., 2020).

Constraints associated with the OCLTT predicted under high temperatures and low levels of water oxygenation (Pörtner *et al*., 2017). In line with this, more degraded waters are expected within or around urban heat islands (Pagliaro and Knouft, 2020; Nelson *et al.*, 2021; Xie *et al*., 2023). We found that heat tolerance was significantly reduced under hypoxia, being particularly notorious during chronic exposure to less extreme temperatures (Fig. 2). Previous studies have quantified upper thermal limits employing warming assays (Beitinger *et al*., 2000; Sunday *et al*., 2014; Brewer *et al*., 2022), which are short-term and might underestimate the sensitivity of different fish species to cope with heat stress. Then, the species are under huge threat if there is a mismatch between the biological response to either long-term or extreme events linked with global warming, having profound ecological consequences (Harris *et al*., 2018). Here, the tolerance landscape approach, based on TDT curves, takes advantage of explicit exposure time consideration (Rezende *et al*., 2014), allowing the acute (i.e. heatwave pulse) and chronic effects (i.e. climatic trends) of heat stress to be properly disentangled (Troia, 2023; Rezende and Carter, 2024), which also have been recently validated for several fish species worldwide (Molina *et al*., 2025). Therefore, our findings align with the evidence of increased thermal sensitivity *z* with hypoxia, and the disproportionately larger effects of oxygenation on heat tolerance during prolonged assays mirror the results obtained in two freshwater amphipod species (Verberk *et al*., 2023), suggesting that this may constitute a more general response. We support the notion that larger fish, with presumably lower mass-specific metabolism, exhibit higher heat tolerance than smaller ones, all else being equal.

The sylurid *T. areolatus* is a native and ubiquitous species from the southwest Andes riverine system, and with a broad geographical distribution from 31 to 48°S covering ~1800 km of geographical distribution (Alò *et al*., 2024), making this species a biological reference to extrapolate this physiological pattern on a geographical scale found in the Maipo hydrological basin. While we have limited information to determine which specific environmental factors might explain the difference in size distribution across populations of *T. areolatus*, the lower body mass and condition state observed in the urban and post-urban sites versus the reference site (Table 1) support the notion that environmental degradation partly accounts for these differences. Changes in the physicochemical characteristics of the Maipo hydrological basin have been observed as a result of anthropogenic activities, land use changes and domiciliary pollution (Chiang *et al*., 2014; Peña-Guerrero *et al*., 2020; Wilkinson *et al*., 2022), with potentially synergistic effects with other stressors such as temperature (Cañedo-Argüelles *et al*., 2013; Cañedo-Arguelles *et al*., 2016). Reduced oxygenation around urban, industrialized and agricultural areas could impact wild populations of *T. areolatus* (Habit *et al*., 2006; Orrego *et al.*, 2009; Orrego *et al.*, 2019; Chiang *et al.*, 2011a), making them more susceptible to heat stress, as suggested by our simulations (Figs 4 and 5). Despite the abundance and wide geographic distribution of *T. areolatus* (Campos, 1998; Habit *et al*., 2007) and its reported resilience to less favourable environmental conditions (Chiang *et al*., 2012a, Chiang *et al.*, 2011a; Chiang *et al.*, 2011b). In this context, *T. areolatus* exhibits substantial plasticity that should be considered when evaluating the effects of different stressors (Chiang *et al.*, 2011a; Chiang *et al.*, 2012a). Therefore, our analytical approaches, in line with previous evidence (Rezende and Carter, 2024; Molina *et al*., 2025), which explicitly account for the chronic effects of heat stress, may reveal physiological and life history responses that are otherwise masked. In line with physiological proxies such as hepatic EROD activity and biochemical responses of detoxification, these have been employed with *T. areolatus* as indicators of exposure to chemical stressors (Chiang *et al.*, 2011a; Orrego *et al.*, 2019; Ali *et al.*, 2020). They exhibit seasonality in metabolic and reproductive baseline values, which may indicate a high induction threshold of this response (Chiang *et al*., 2012b). Different health status indexes (condition factor, gonad somatic and liver somatic indexes) also showed seasonal and high inter-individual variability (Chiang *et al*., 2011b, 2012a), reinforcing the idea that environmental degradation plays a role and negatively impacts these populations. Thus, our work aims to bridge the gap between the physiological impact of environmental degradation on freshwater systems and mechanistic thermal ecology, thereby enhancing our understanding of the tolerance range of wild fish and their potential capacity to respond to anthropogenic activities and global change.

## Supplementary Material

Web_Material_coaf081

## Data Availability

Data will be made available on request.
