# Supplementary material for: Effect of body size on heat tolerance of a freshwater catfish (Trichomycterus areolatus)
Source: Conserv Physiol. 2025 Nov 25;13(1):coaf081. doi: 10.1093/conphys/coaf081 (PMC12649744; doi:10.1093/conphys/coaf081)
Supplement: Web_Material_coaf081 [file web_material_coaf081.pdf]

Supplementary Online Material

for

Effect of body size on heat tolerance of a freshwater catfish (*Trichomycterus areolatus*)

Daniel Avilés-Hernández, Cristián A. Zamora, Ian Calderon-Castro, D. Patricio Carrizo,

Gustavo Chiang, Enrico L. Rezende, Mauricio J. Carter

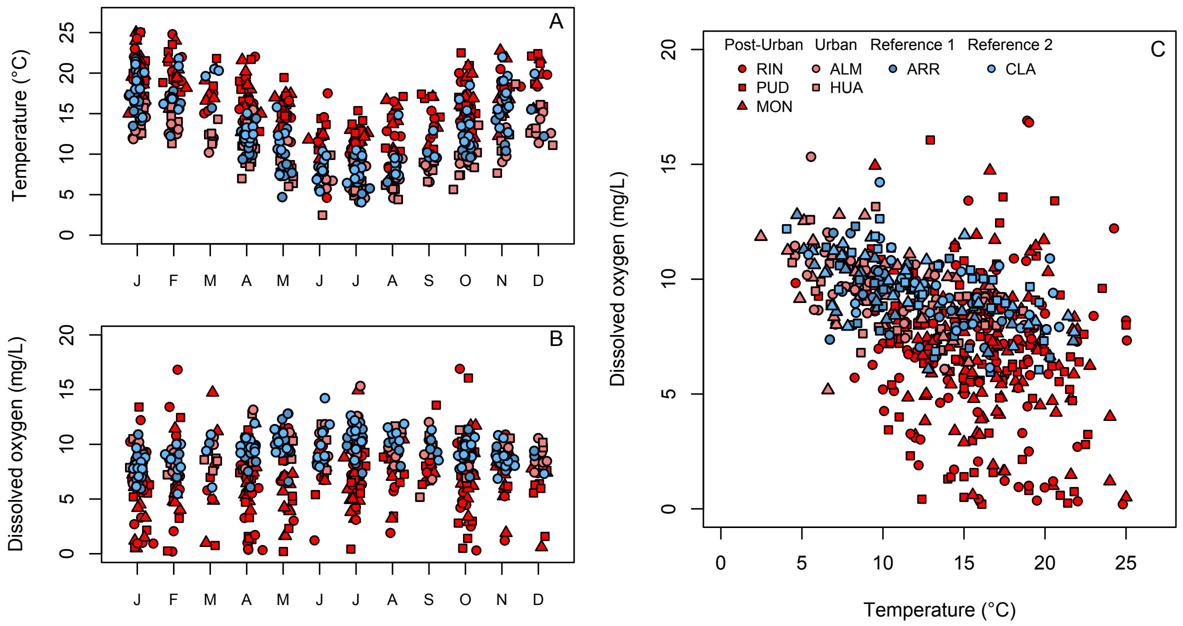

Figure S1: The historical temperature and dissolved oxygen concentration pattern from seven monitoring stations (1985-2022) belong to the Chilean monitoring water quality survey (DGA: https://dga.mop.gob.cl). Descriptions about monitoring stations are detailed in Table S1.

Table S1: Environmental data from monitoring stations used to describe the four study sites. Data are presented as mean and range Data were extracted from Dirección General de Aguas (https://snia.mop.gob.cl/BNAConsultas/reportes) and represent data from a range since January 1985 to December 2022 (fragmented data).

Site	code	DGA name	Latitude	Longitude	Altitude (msnm)	Temperature (C°)	DO (mg/L)	pH	Conductivity (µS/cm)	Period
Reference 1	CLA	Rio Clarillo antes junta Río Maipo	33° 38' 50''	70° 37' 50''	640	14.0 (4.05 -28.81)	9.64 (14.22 - 5.47)	8.46 (12.18 - 6.61)	209 (270 - 134)	1995-2021
Reference 2	ARR	Estero Arrayan en La Montosa	33° 19' 32''	70° 27' 22'	880	11.53 (21.7 - 4.70)	9.17 (12.8 - 5.95)	7.88 (9.48 - 7.02)	276 (379 - 137)	1997-2019
Urban	ALM	Río Mapocho en Los Almendros	33° 22' 14''	70° 27' 04''	866	10.3 (19.3 - 2.47)	9.58 (15.33 - 5.17)	7.7 (10.96 – 5.68)	288 (883 – 116)	1998-2021
	HUA	Río Mapocho después de junta con Estero Hualtatas	33° 22' 16''	70° 31' 15''	792	12.23 (18.64 – 7.12)	8.77 (10.4 – 7.62)	8.12 (8.36 – 7.92)	405 (539 – 222)	2018-2021
Post-urban	RIN	Río Mapocho Rinconada de Maipú	33° 29' 60''	70° 49' 08''	440	15.9 (25.0 – 9.0)	4.31 (10.86 – 0.2)	7.49 (8.46 – 6.50)	1253 (2213 – 188)	1985-2018
	PUD	Río Mapocho en pte Pudahuel	33° 26' 14''	70° 49' 08''	450	14.34 (22.7 – 4.61)	6.79 (16.9 – 0.25)	7.69 (9.38 – 5.86)	1189 (2159 – 379)	1985-2018
	MON	Río Mapocho en El Monte	33° 40' 40''	70° 58' 21''	278	16.71 (25.1 – 9.29)	7.97 (16.8 – 3.24)	7.89 (11.8 – 6.87)	1431 (1930 – 909)	1990-2022

Table S2: Pairwise comparison of morphological fish measurement from *T. areolatus* populations collected along of the sample site in Maipo river basin.

	Estimate (SE)	t ratio (df=236)	p value
**Fork length**			
Urban vs Post-urban	**1.590 (0.333)**	**4.780**	**<0.0001**
Urban vs Reference 1	0.164 (0.314)	0.521	0.9539
Urban vs Reference 2	-0.359 (0.341)	-1.051	0.7196
Post-urban vs Reference 1	**-1.426 (0.351)**	**-4.064**	**<0.001**
Post-urban vs Reference 2	**-1.949 (0.375)**	**-5.191**	**<0.0001**
Reference 1 vs Reference 2	-0.522 (0.359)	-1.455	0.4667
**Mass**			
Urban vs Post-urban	**1.793 (0.359)**	**4.999**	**<0.0001**
Urban vs Reference 1	**1.459 (0.339)**	**4.309**	**0.0001**
Urban vs Reference 2	0.558 (0.368)	1.518	0.4285
Post-urban vs Reference 1	-0.334 (0.378)	-0.882	0.8144
Post-urban vs Reference 2	**-1.235 (0.405)**	**-3.050**	**0.0135**
Reference 1 vs Reference 2	-0.901 (0.387)	-2.327	0.0948
**Condition Factor (K)**			
Urban vs Post-urban	-0.008 (0.043)	-0.194	0.9974
Urban vs Reference 1	**0.221 (0.041)**	**5.145**	**<0.0001**
Urban vs Reference 2	**0.259 (0.044)**	**5.853**	**<0.0001**
Post-urban vs Reference 1	**0.229 (0.045)**	**5.031**	**<0.0001**
Post-urban vs Reference 2	**0.267 (0.048)**	**5.492**	**<0.0001**
Reference 1 vs Reference 2	0.038 (0.046)	0.835	0.8426

Table S3: Simulating mortality, cumulative mortality estimated using 118 days of highly detailed field records from three sites, which reflect the Reference, Urban, and Post-urban environmental context**.** Min wt: warming tolerance at minutes, Temp at min: temperature at minutes & Time at min: time at minutes

	El Clarillo Reference site			Las Hualtatas Urban site			El Monte PostUrban site		
Day	Min wt	Temp at min	Time at min	Min wt	Temp at min	Time at min	Min wt	Temp at min	Time at min
1	11,27	20,10	123	8,45	23,84	99	5,93	19,70	862
2	10,42	20,94	125	8,32	24,07	82	5,72	20,75	413
3	10,13	21,21	130	6,68	25,59	104	4,10	22,29	442
4	9,87	21,53	117	9,51	22,67	124	4,95	22,73	143
5	11,35	20,13	102	8,80	23,29	147	4,62	20,98	884
6	12,06	19,08	185	7,53	24,58	141	2,64	22,94	900
7	10,09	21,24	133	6,49	25,70	120	2,25	23,59	717
8	10,22	21,28	98	7,88	24,34	114	2,16	23,42	899
9	9,47	21,89	124	9,29	22,62	210	2,05	24,05	573
10	8,83	22,55	121	9,79	21,90	329	1,97	24,61	375
11	8,09	23,23	135	7,69	24,53	114	1,70	24,43	556
12	8,94	22,44	121	8,54	23,71	108	1,83	24,12	648
13	8,40	22,89	141	8,93	23,34	103	1,88	23,76	853
14	8,09	23,23	136	8,50	23,78	102	1,78	23,80	900
15	8,46	22,88	131	9,35	22,69	163	1,70	23,95	843
16	7,66	23,74	116	10,28	21,30	409	1,47	24,12	890
17	9,66	21,63	141	9,71	22,30	172	1,56	23,89	1002
18	8,07	23,28	128	7,23	25,07	96	2,03	23,56	893
19	7,63	23,74	123	5,52	26,75	103	2,27	23,32	889
20	10,58	20,79	125	5,45	26,80	108	2,34	23,14	983
21	7,63	23,73	126	5,84	26,37	116	2,14	23,49	861
22	6,59	24,84	110	5,77	26,65	77	2,02	24,28	479
23	6,67	24,68	127	4,89	27,35	108	1,88	23,72	881

	El Clarillo Reference site			Las Hualtatas Urban site			El Monte PostUrban site		
Day	Min wt	Temp at min	Time at min	Min wt	Temp at min	Time at min	Min wt	Temp at min	Time at min
24	6,68	24,75	111	5,25	27,03	102	1,71	23,88	892
25	6,52	24,85	125	5,06	27,31	85	1,74	23,76	961
26	7,51	23,84	127	6,05	26,13	124	1,92	24,53	420
27	6,65	24,69	128	8,29	23,72	172	1,98	23,66	852
28	6,16	25,20	124	6,51	25,68	120	1,69	23,90	893
29	5,65	25,67	136	7,70	24,34	164	1,54	24,02	916
30	5,90	25,44	130	6,04	26,17	115	1,52	23,99	957
31	6,94	24,39	134	6,28	25,91	122	1,70	23,76	1006
32	6,14	25,23	122	8,73	23,27	177	1,96	23,39	1100
33	6,41	24,95	124	8,70	23,32	168	1,60	24,84	426
34	6,36	25,02	121	7,59	24,56	131	2,02	23,45	995
35	6,27	25,03	138	7,96	24,08	162	1,84	23,83	836
36	7,20	23,94	184	8,27	23,66	202	1,62	23,97	894
37	5,17	26,26	112	8,26	23,56	253	1,51	24,09	885
38	5,96	25,38	129	6,50	25,65	131	1,52	24,02	937
39	5,59	25,74	132	6,63	25,47	145	1,34	24,33	832
40	5,56	25,83	118	6,59	25,66	107	1,45	25,21	348
41	5,73	25,68	115	5,61	26,68	99	1,80	24,63	428
42	5,88	25,44	135	5,86	26,39	109	1,52	24,41	660
43	6,65	24,76	116	5,65	26,53	123	1,24	24,46	813
44	6,50	24,63	188	8,41	22,76	898	1,41	24,12	937
45	5,55	25,82	124	5,03	27,33	86	1,48	24,09	911
46	6,40	24,99	119	8,24	23,28	456	1,78	23,83	873
47	5,57	25,82	120	5,55	26,73	101	1,89	24,19	578
48	6,14	25,19	132	5,68	26,62	97	1,91	23,71	870
49	6,73	24,64	124	9,55	22,46	173	1,79	24,00	749
50	6,60	24,71	135	6,38	26,05	75	1,63	24,10	795

	El Clarillo Reference site			Las Hualtatas Urban site			El Monte PostUrban site		
Day	Min wt	Temp at min	Time at min	Min wt	Temp at min	Time at min	Min wt	Temp at min	Time at min
51	6,83	24,52	127	7,06	25,32	83	2,67	23,92	371
52	6,70	24,64	128	11,10	20,63	303	2,70	23,84	389
53	6,46	24,90	126	10,91	20,99	218	2,46	24,70	225
54	6,27	25,09	125	9,94	21,99	201	2,63	24,22	296
55	6,22	25,08	139	7,31	25,08	81	2,70	24,42	234
56	5,96	25,45	113	8,60	23,31	210	1,77	24,09	703
57	6,13	25,23	127	9,03	22,67	322	1,43	24,60	610
58	6,06	25,23	140	9,44	22,43	231	1,54	24,20	784
59	5,81	25,65	106	7,63	24,56	122	1,54	24,17	803
60	6,09	25,20	142	9,31	22,43	294	1,57	24,19	768
61	5,83	25,48	137	5,05	27,39	74	1,69	24,02	801
62	5,87	25,48	128	4,69	27,45	134	2,17	24,38	386
63	6,21	25,07	143	6,14	26,15	98	1,35	24,33	825
64	5,96	25,43	119	6,39	25,96	88	1,22	24,53	781
65	6,76	24,68	108	6,34	25,96	97	1,09	24,62	802
66	6,65	24,68	132	7,24	24,95	120	1,06	24,73	751
67	6,45	24,88	131	7,43	24,60	166	1,14	24,48	868
68	6,38	25,02	118	8,07	23,92	178	1,31	24,36	837
69	6,32	24,95	147	7,23	24,74	186	1,21	24,48	817
70	6,00	25,29	142	9,28	22,40	331	1,05	24,67	793
71	6,13	25,28	114	6,89	25,32	115	0,87	24,81	823
72	5,97	25,38	128	5,37	26,91	102	0,78	24,73	965
73	6,04	25,39	112	9,24	22,58	252	0,82	24,82	854
74	6,91	24,48	120	6,38	25,65	168	0,83	24,65	979
75	7,13	24,27	117	6,22	26,06	102	1,78	24,58	456
76	7,83	23,59	113	6,38	25,95	93	5,21	21,08	485
77	8,23	23,14	125	6,96	25,35	95	1,64	24,36	627

	El Clarillo Reference site			Las Hualtatas Urban site			El Monte PostUrban site		
Day	Min wt	Temp at min	Time at min	Min wt	Temp at min	Time at min	Min wt	Temp at min	Time at min
78	8,08	23,26	129	6,50	25,79	99	1,21	24,31	946
79	7,81	23,56	124	6,14	26,39	62	1,20	24,31	957
80	7,99	23,39	121	8,75	23,36	141	1,27	24,36	866
81	8,32	22,98	141	8,33	23,66	180	1,30	24,22	947
82	7,46	23,87	134	5,05	27,31	87	1,45	24,10	927
83	7,28	24,06	130	5,22	27,07	99	1,36	24,34	816
84	7,38	23,98	126	5,83	26,49	94	1,17	24,48	848
85	7,01	24,32	133	7,07	25,26	92	1,28	24,29	908
86	7,24	24,11	126	8,03	24,43	70	1,33	24,40	788
87	7,25	24,12	124	7,35	24,84	121	1,51	24,16	827
88	7,33	24,02	127	10,93	20,82	289	1,61	24,12	786
89	7,94	23,44	121	9,20	23,03	112	1,45	24,31	771
90	8,32	22,97	143	11,24	20,03	742	1,51	24,05	919
91	8,06	23,24	139	10,08	22,10	123	1,43	24,35	755
92	7,74	23,60	128	10,62	20,86	493	1,23	24,53	772
93	7,83	23,61	109	11,07	20,35	555	1,25	24,38	867
94	8,19	23,11	139	10,76	21,12	225	1,38	24,31	817
95	8,45	22,92	124	9,25	22,96	116	1,28	24,48	771
96	8,48	22,86	131	7,27	25,65	29	1,33	24,24	911
97	8,55	22,72	146	10,87	21,16	165	1,40	24,24	855
98	8,25	23,10	126	10,14	22,39	62	1,42	24,33	772
99	8,98	22,27	153	8,85	24,22	21	1,40	24,34	784
100	9,20	22,15	127	11,79	19,69	496	1,33	24,43	771
101	9,03	22,30	133	11,38	20,69	155	1,32	24,36	825
102	8,77	22,56	133	5,13	27,08	117	1,60	23,99	893
103	8,81	22,48	141	5,09	27,12	115	1,82	23,81	867
104	9,24	22,00	154	6,61	25,84	72	1,58	24,14	793

	El Clarillo Reference site			Las Hualtatas Urban site			El Monte PostUrban site		
Day	Min wt	Temp at min	Time at min	Min wt	Temp at min	Time at min	Min wt	Temp at min	Time at min
105	9,58	21,77	129	6,48	26,01	67	1,67	24,02	815
106	9,44	21,95	119	10,60	21,36	193	1,75	23,94	818
107	9,68	21,70	121	4,83	27,56	82	1,65	24,12	760
108	9,89	21,46	127	6,53	25,49	171	1,56	24,24	741
109	10,09	21,25	130	4,46	27,86	93	1,57	24,17	787
110	9,73	21,62	126	8,58	23,82	80	1,63	24,03	843
111	9,64	21,75	118	4,67	27,79	72	1,62	24,19	741
112	10,23	21,08	137	10,65	21,61	106	1,62	24,10	797
113	10,82	20,33	179	5,85	26,67	63	1,63	24,17	742
114	11,64	19,65	142	6,25	26,15	79	1,60	24,12	793
115	12,47	19,07	92	7,47	24,86	92	1,60	24,14	786
116	12,19	19,21	116	6,58	25,82	80	1,83	23,88	806
117	14,08	17,19	147	7,35	24,97	94	2,02	23,58	890
118	13,96	17,05	233	7,94	24,34	101	2,19	23,69	695
